# Synthesis and Characterization of Cellulose-Based Hydrogels to Be Used as Gel Electrolytes

**DOI:** 10.3390/membranes5040810

**Published:** 2015-11-27

**Authors:** Maria Assunta Navarra, Chiara Dal Bosco, Judith Serra Moreno, Francesco Maria Vitucci, Annalisa Paolone, Stefania Panero

**Affiliations:** 1 Department of Chemistry, Sapienza University of Rome, Piazzale Aldo Moro 5, Rome 00185, Italy; E-Mails: chiara.dalbosco@virgilio.it (C.D.B.); jusebcn@gmail.com (J.S.M.); stefania.panero@uniroma1.it (S.P.); 2 CNR-ISC, UOS La Sapienza, Piazzale Aldo Moro 5, Rome 00185, Italy; E-Mails: francesco.vitucci@roma1.infn.it (F.M.V.); annalisa.paolone@roma1.infn.it (A.P.)

**Keywords:** cellulose hydrogels, gel electrolyte membranes, swelling behavior, ionic conductivity and diffusivity

## Abstract

Cellulose-based hydrogels, obtained by tuned, low-cost synthetic routes, are proposed as convenient gel electrolyte membranes. Hydrogels have been prepared from different types of cellulose by optimized solubilization and crosslinking steps. The obtained gel membranes have been characterized by infrared spectroscopy, scanning electron microscopy, thermogravimetric analysis, and mechanical tests in order to investigate the crosslinking occurrence and modifications of cellulose resulting from the synthetic process, morphology of the hydrogels, their thermal stability, and viscoelastic-extensional properties, respectively. Hydrogels liquid uptake capability and ionic conductivity, derived from absorption of aqueous electrolytic solutions, have been evaluated, to assess the successful applicability of the proposed membranes as gel electrolytes for electrochemical devices. To this purpose, the redox behavior of electroactive species entrapped into the hydrogels has been investigated by cyclic voltammetry tests, revealing very high reversibility and ion diffusivity.

## 1. Introduction

Hydrogels derived from natural polymers, especially polysaccharides, are very interesting materials since they find application in many fields (agriculture, tissue engineering, drug delivery, biosensors, *etc.*) [1] with the advantage of being prepared starting from environmentally-friendly, renewable, and low cost raw materials. Among polysaccharides, cellulose is the most abundant one, available worldwide and it combines hydrophilicity with good mechanical properties. Both of these competitive characteristics are due to the numerous hydroxyl groups that interact by hydrogen bonds preferentially with water (amorphous domains) or with hydroxyl groups of adjacent polymer chains (crystalline domains) [2]. The complex system of hydrogen bonds between hydroxyl groups (supramolecular structure) contributes to both cellulose’s mechanical strength and its insolubility in water and in the major part of solvents [2].

Intense research has focused on the dissolution of cellulose are providing continuous improvements of efficiency in solvents such as NaOH/urea aqueous solutions and similar systems (*i.e.*, NaOH/urea/thiourea, NaOH/polyethylene glycol, LiOH/urea) [3,4,5,6,7,8,9,10,11,12], which are non-derivatizing, inexpensive and non-toxic cellulose solvents. The effort for a systematic study is complicated by the variability of cellulose, whose characteristics change with its source and batches. Therefore, solubilization parameters have to be adjusted depending on the particular type of cellulose. 

In the literature characterizations of many kinds of hydrogels prepared from different types of cellulose, alone or mixed with its derivatives [13,14,15,16], such as lignin [17], chitin [18], or polyvinyl alcohol [19], are reported. The synthesis of cellulose-based hydrogels generally consists of two steps [1,13,14,15,16,17,18,19]: (i) solubilization of cellulose fibers or powder; and (ii) chemical and/or physical crosslinking, in order to obtain a three-dimensional network of hydrophilic polymer chains, which is able to absorb and retain a significant amount of water. 

By tuning the crosslinker and cellulose concentrations, it is possible to optimize the hydrogel mechanical properties and swelling capabilities.

Thanks to these recent developments and due to the growing importance of green chemistry, versatile materials derived from cellulose, such as hydrogels and aerogels, have raised renewed interest in the field of electrochemical devices involving energy generation and storage [20,21,22]. The latest application concerns the use of cellulose, or its derivatives, as environmentally-friendly and economic non-aqueous gel polymer electrolytes for lithium and sodium ion batteries [23,24,25]. Nevertheless, cellulose hydrogels could also have interesting applications as aqueous gel polymer electrolytes, for example in aqueous electric double-layer capacitors and dye-sensitized solar cells [20,26]. Cellulose has also been considered as possible starting materials for proton exchange membranes (PEMs) in PEM fuel cells (FCs) [27], as well as electrode components in some types of FCs and supercapacitors [26,27,28,29,30]. Very recently, electricity and H_2_ generation from hemicellulose by sequential fermentation and microbial fuel/electrolysis cells has been reported [31]. To the best of our knowledge, electrochemical data on pure cellulose hydrogels are almost absent in the literature, or limited to particular hybrid hydrogels and electrolytes [20,32].

In this framework, the present work reports on hydrogels obtained exclusively from unsubstituted celluloses crosslinked with epichlorohydrin (ECH) through optimized steps to achieve both the best solubilization and crosslinking. A detailed physical chemical characterization on both molecular and structural levels, as well as on the mechanical properties of the proposed hydrogel membranes, will be here presented. Originally, swelling capability and electrochemical properties, investigated in terms of ion conduction ability and redox behavior of absorbed electroactive species, will be discussed, as well, to demonstrate the potential of the cellulose hydrogel membranes as ion-conducting, gel polymer electrolytes.

## 2. Results and Discussion

FTIR spectroscopy has been extensively used in the study of cellulose materials, since it allows for obtaining direct information on the main modifications that occur in cellulose during various chemical and physical treatments [33].

**Figure 1:**
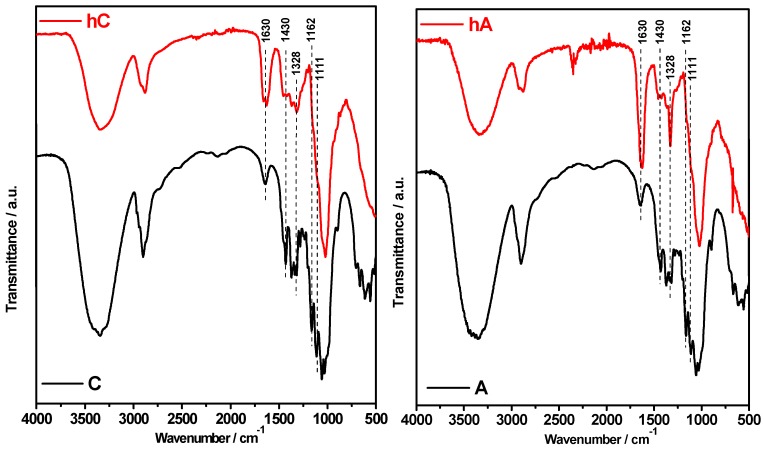
FTIR spectra of the C and A starting celluloses compared with their hydrogels (hC and hA, respectively).

ATR-FTIR spectra of the hydrogels (hC and hA) compared with their pristine cellulose (C and A) are shown in Figure 1.

With respect to pristine celluloses, in the spectra of the hydrogels we can observe that:

(i) the band at 1430 cm^−1^ (CH_2_ symmetric bending) [17,34,35] strongly decreased in intensity; (ii) the band at 1328 cm^−1^ (C-C and C-O cellulose skeletal vibrations) [33,36] increased, especially in the spectrum of hA; and (iii) the bands at 1162 and 1111 cm^−1^ disappear. In addition, by comparing the spectra of the hydrogels it can be observed that the band at 1630 cm^−1^ (adsorbed water) [37] is more intense in hA than in hC.

**Figure 2:**
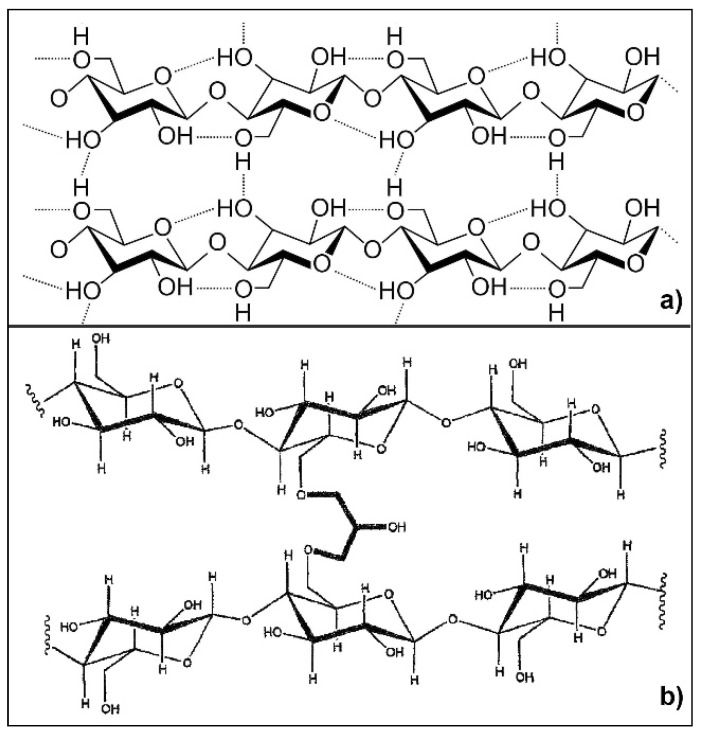
(**a**) Scheme of cellulose physical crosslink (intra- and inter-molecular hydrogen bonds); and (**b**) scheme of cellulose chemical crosslink (ether bonds).

Spectral modification in the region 1500–899 cm^−1^ is imputable to an alteration of cellulose crystalline organization [24] due to the alkali treatment [6,10,38]. In particular, point (iii) indicates that a crystal transformation from cellulose I to cellulose II has occurred in the hydrogels [28,39]. Moreover, the decreased intensity of the band at 1430 cm^−1^ (known as the “crystallinity band”) stated at point (i) reflects a decrease of the crystallinity degree of cellulose [17,24]. Point (ii) can be related to the occurred chemical crosslinking of cellulose with ECH producing ether-based linkage, which is an indirect proof of the effectiveness of the solubilization step. With the adopted cellulose and ECH concentrations the obtained hydrogels are expected to be both chemically and physically crosslinked [14], as depicted in Figure 2. At room temperature (RT) the reaction of ECH with the hydroxyl groups of cellulose is in competition with the cellulose chains entanglement (physical crosslinking). Since the physical crosslinking is related to the cellulose DP (the higher the DP, the shorter the gelation time at RT), hC is thought to be more physically cross-linked than hA. Indeed, contrary to cellulose “A”, the beginning of physical gelation in the case of starting material “C” has been observed already during the reaction with ECH. This is consistent with the increased intensity of the band at 1630 cm^−1^ (adsorbed water) [37] in hA spectrum, reflecting a higher hydrophilic character of hA than hC due to the prevalence of the chemical crosslinking on the physical one. It is known that the etherification reaction introduces local hydrophilic domains for water sorption and prevents adjacent cellulose chains from establishing intermolecular hydrogen bonds.

**Figure 3:**
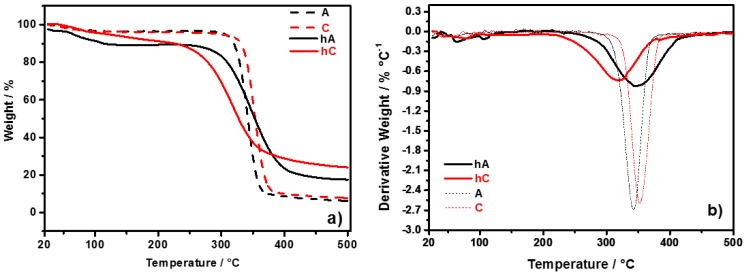
(**a**) TGA curves of C and A celluloses, and of their hydrogels (hC and hA, respectively); (**b**) First derivative of weight curves.

Thermal behavior of the synthesized hydrogels, compared with starting celluloses, has been evaluated by means of TGA response, reported in Figure 3. Figure 3a shows the TGA curves of the pristine celluloses (C and A) in comparison with those of the relative vacuum-dried hydrogels (hC and hA). In Figure 3b the corresponding first derivative curves are reported, from which the onset temperature (T_onset_) of degradation and the decomposition temperature (T_d_) have been determined, the former by the point of intersection of tangents to two branches of the curve, the latter from the peak minimum.

The thermal behavior of C and A is similar regarding the water content (weight loss at 100 °C is *ca.* 3.5%) and the onset temperature (*i.e.*, 290 °C), while the thermal decomposition temperatures (T_d_) of C and A are 352 °C and 342 °C, respectively. This difference can be related to the lower DP of A with respect to that of C. The thermal behavior of the hydrogels is different between each other and with respect to their pristine celluloses. The weight loss at 100 °C is higher in hA (8%) than in hC (4%), this result indicating a more hydrophilic character of hA compared with hC (as also arised from FTIR spectra). T_onset_ and T_d_ are respectively 220 °C and 320 °C for hC, 245 °C and 347 °C for hA. A comparison with the pristine celluloses shows that: (i) both the hydrogels have a lower T_onset_, but the difference is greater for hC (70 °C) than for hA (45 °C); (ii) T_d_ is 32 °C lower for hC respect to C, and 5 °C higher for hA respect to A. The decrease of T_onset_ in the hydrogels is due to the lower crystallinity degree of cellulose [28,40] (see discussion on FTIR spectra). The thermal stability of hA is improved by the chemical crosslinking (proved by FTIR investigation), which limits the decrease of T_onset_ and raises T_d_.

**Figure 4:**
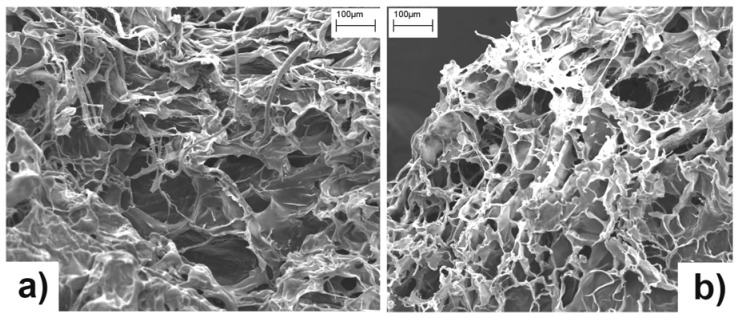
SEM micrographs of freeze-dried hC (**a**) and hA (**b**) hydrogels.

SEM images of freeze-dried hydrogels are shown in Figure 4. Both samples appear characterized by a sponge-like surface morphology, with the presence of open channels mixed with smoother interconnecting domains, probably due to the growth of relatively large ice crystals during the freeze-drying from water [41].

**Figure 5:**
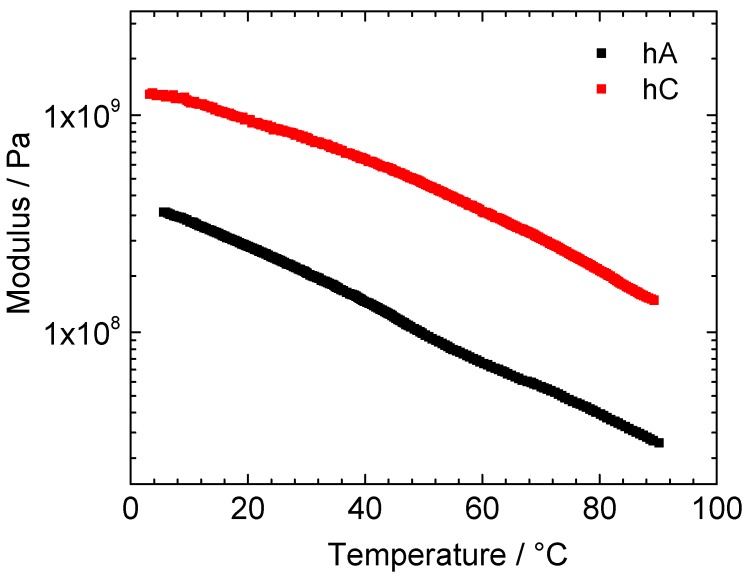
Temperature dependence of the storage modulus of vacuum-dried hA (black) and hC (red).

In Figure 5 it is shown the temperature dependence of the storage modulus of vacuum-dried hC and hA samples.

The modulus of hC is higher than that of hA in the whole temperature range. At room temperature 9.1·10^8^ Pa and 2.3·10^8^ Pa were obtained for hC and hA, respectively, these values being typical for crosslinked tridimensional polymer materials. Increasing the temperature, both samples show a monotone modulus decrease until the maximum investigated temperature (about 90 °C).

**Figure 6:**
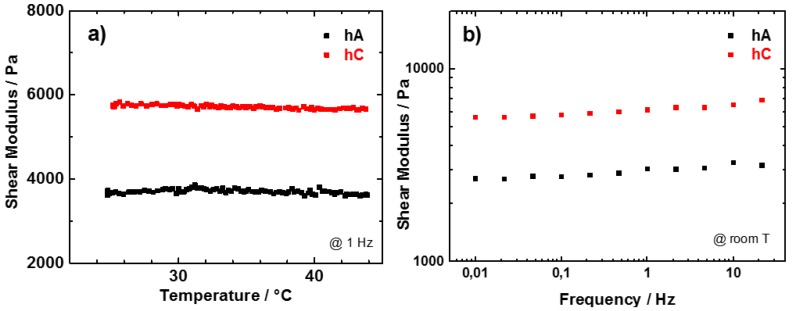
(**a**) Temperature; (**b**) frequency dependence of the shear modulus of pristine hA (black) and hC (red).

The hydrogels, as obtained after the synthesis, have been characterized in terms of shear modulus, measured in the temperature range between 23 and 44 °C and reported in Figure 6a. In this range the mass loss due to the evaporation of water is quite small and should not strongly influence the modulus values. As for the dry samples, hA presents a lower modulus value with respect to hC, possibly attributed to the higher water content in hA with respect to hC. The shear modulus of both the hydrogels is practically independent on temperature.

In order to study the viscoelastic response of the hydrogels, shear modulus measurements have been performed also at room temperature as a function of frequency. Measurements performed between 0.01 and 22 Hz are reported in Figure 6b. For both hC and hA the shear modulus depends weakly on the frequency. The small increase of the shear modulus as the frequency increases is typical of all polymers [42]. The shear modulus measured at RT and at 1Hz are ~3000 Pa for hA and ~6000 Pa for hC. These values are sensibly higher than those measured in similar conditions by Chang *et al.* [14] that reported shear moduli between 30 and 800 Pa for hydrogel obtained with the same cellulose concentration by freezing methods. This difference could be ascribed to the different type of cellulose and to the different crosslink of the polymers.

**Figure 7:**
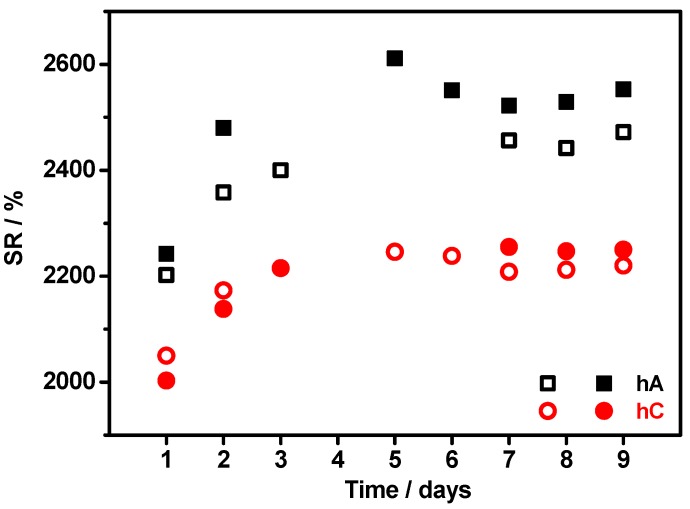
Time dependence of the swelling ratio of hC and hA immersed in 0.5 M Na_2_SO_4_ aqueous solution at RT (for each hydrogel determination has been carried out on two different samples).

The swelling ratio (SR) of hC and hA immersed in 0.5 M Na_2_SO_4_ aqueous solution at RT has been monitored on time and reported in Figure 7. Determination has been carried out on two different samples taken from each hydrogel.

The hydrogels reach a saturation state within three to five days, thereafter they hold the absorbed solution without leaking nor drying for a long time (at least six months), when kept in a covered container. To be noticed that, even after drying, the membrane is able to reversibly absorb the swelling solution. As expected from SEM observations, both the hydrogels present a high swelling capability, being it 2233 ± 19% for hC and 2504 ± 46% for hA (calculated as average of the SR values from 6th day on). The higher SR of hA can be related to a wider extension of the chemical crosslinking with respect to hC (see FTIR results): this implies a higher number of oxygen atoms resulting from the etherification reaction, which are available to interact with water through hydrogen bond. The variability of the SR is higher in hA than in hC because of the difficult to manipulate samples of hA, which were softer and stickier than those of hC.

These SR values are 50%–70% higher than that determined at RT in distilled water by Chang *et al.* [14] on hydrogels prepared with the freezing method and the same cellulose concentration. This difference is even underestimated, since the swelling ratio is known to increase with a decrease of the ionic strength of the solution [15].

**Figure 8:**
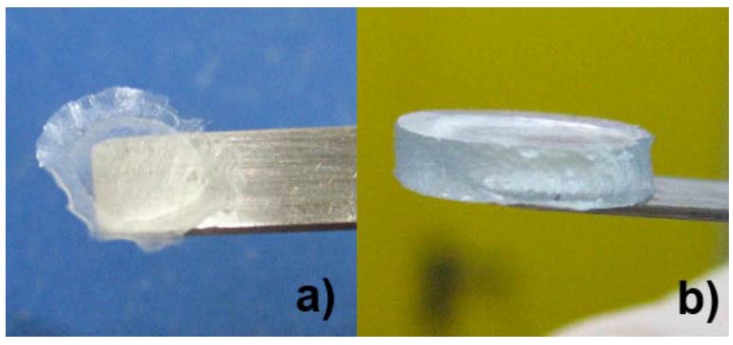
(**a**) Dried state of hC; and (**b**) swollen state of hC.

Superabsorbent properties of the hydrogels are evident from Figure 8, which shows a sample of hC vacuum-dried for 24 h (Figure 8a) and then swelled for 24 h in a 0.5 M Na_2_SO_4_ aqueous solution (Figure 8b). The average thicknesses of hC are 60 ± 11 µm in the dry state and 2.1 ± 0.7 mm when swelled.

**Figure 9:**
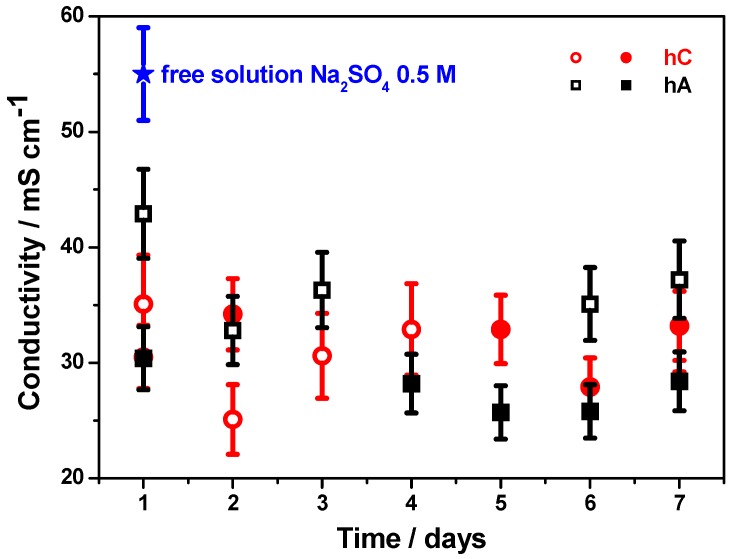
Ionic conductivity values at room temperature recorded on time for hA and hC swelled for 24 h in 0.5 M Na_2_SO_4_ aqueous solutions (for each hydrogel determination has been carried out on two different samples).

Ion-conducting ability of the hydrogel membranes has been proved by electrochemical impedance spectroscopy performed on hC and hA samples swelled for 24 h in 0.5 M Na_2_SO_4_ aqueous solution. Conductivity values as a function of time at RT are reported in Figure 9.

Both membranes show very high RT ionic conductivity, approaching the value measured for the 0.5 M Na_2_SO_4_ swelling solution (0.055 ± 0.004 S cm^−1^). Overall, the differences on time and between samples are not statistically significant since they are within the experimental errors. This result indicates that both hC and hA have adequate highly open porous structures with well interconnected pores which allows an agile migration of the ions under the applied electric field.

**Figure 10:**
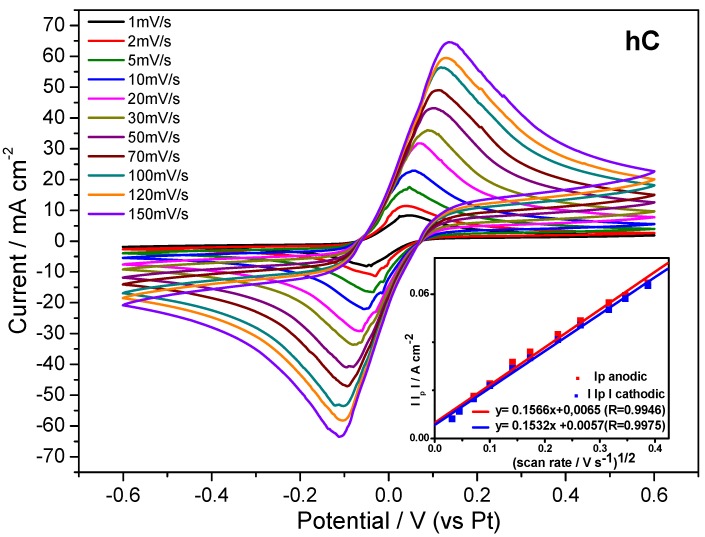
Cyclic voltammetry tests on hC swelled at RT in 0.25M K_4_[Fe(CN)_6_]3H_2_O/K_3_[Fe(CN)_6_] aqueous solution registered at different scan rates, and (INSET) the plot of peak current density *vs.* square root of potential scan rate.

This evidence has suggested to investigate the electrochemical behavior of the highly-reversible redox system Fe(CN)_6_^4−^/Fe(CN)_6_^3−^ trapped in the cellulosic polymer matrix. Thus, cyclic voltammetry analysis has been carried out on hydrogels swollen in 0.25 M K_4_[Fe(CN)_6_]3H_2_O/K_3_[Fe(CN)_6_] aqueous solution. The cyclic voltammetry results on hC, which has been chosen as the most promising sample for its better mechanical properties, are reported in Figure 10, along with the plot of the peak current density as a function of square root of the potential scan rate.

The obtained linear relationship, expected for a typical Nernstian, free solution, indicates that the redox reaction is diffusion controlled also inside the polymer matrix. Indeed, from Figure 10 is evident that the ratio of the anodic and cathodic current peak heights is close to one. This result, along with the peak potential spacing (ΔE_p_) ≤ 59 mV at each potential scan rate, as expected for a fast, reversible, one-electron transfer at 298 K, means that the redox process is reversible within a wide potential scan rate range, the gelled electroactive electrolyte showing a Nernstian behavior. As expected from these results, the diffusion coefficient of the redox couple Fe(CN)_6_^4−^/Fe(CN)_6_^3−^ calculated from hC voltammetries by applying the Randles-Sevcik equation (D_hc_ = 5.31 × 10^−6^ ± 3.15x10^−7^ cm^2^ s^−1^) approaches the reference value [43], typical of aqueous electrolyte solutions.

## 3. Experimental Section

### 3.1. Reagents.

NaOH (>97%), H_2_SO_4_ (96%), and Na_2_SO_4_ (99.5%) were purchased from Carlo Erba (Milan, Italy) and used without further purification. Urea (100%), epichlorohydrin (≥99%, ECH) and C6288 cellulose (fibers) were purchased from Sigma Aldrich (St. Louis, MO, USA). Microcrystalline cellulose Avicel^®^ PH-101 (average molecular weight (MW) and degree of polymerization (DP): 3.9 × 10^4^ and 230, respectively) was purchased from Fluka (St. Louis, MO, USA). K_4_[Fe(CN)_6_]3H_2_O (99%) and K_3_[Fe(CN)_6_] (99%) were purchased from Merck (St. Louis, MO, USA).

C6288 and Avicel^®^ PH-101 celluloses have been labeled as C and A, respectively.

### 3.2. Synthesis of cellulose-based hydrogels.

The proposed synthesis refers to the procedure already published by C. Chang *et al.* [14], bringing about some important changes. Two types of hydrogels have been prepared, starting from a cellulose with lower or higher MW and DP, Avicel^®^ PH-101 and C6288 celluloses, respectively. In detail, both types of cellulose have been solubilized in an aqueous solution of 6 wt.% NaOH/4 wt.% urea, consisting of 6 g of NaOH, 4 g of urea and 90 ml of ultrapure water (MilliQ water, Millipore). Solubilization occurred at low temperature (between −13 and −15 °C) by several freezing-thawing cycles up to the achievement of a transparent 4 wt.% cellulose solution. The solution was then poured into a Petri dish and ECH 5 vol.% was added dropwise at room temperature (RT) under magnetic stirring. After 1 h, the Petri dish was placed into freezer at −30 °C for 20 h. After thawing the solid at RT, the hydrogel was obtained within 24 h. The obtained hydrogel membranes were washed in ultrapure water (MilliQ water, Millipore), by means of an ultrasonic bath, in order to remove the residual reagents. The washing steps finished when the last washing solution reached pH 7.

Hydrogels derived from starting C or A cellulose have been labeled as hC or hA, respectively.

### 3.3. Characterizations.

Fourier Transform Infrared spectroscopy (FTIR). FTIR spectra were recorded using a Bruker ALPHA spectrometer (Billerica, MA, USA) in the 4000–400 cm^−1^ frequency range, at a resolution of 4 cm^−1^. Vacuum-dried hydrogel samples were analyzed by 300 scans in attenuated total reflectance (ATR) mode, while cellulose powder and fibers were analyzed in KBr discs. 

Thermogravimetric analysis (TGA). Thermal stability of the hydrogels was studied for both cellulose powders and dried hydrogels by TGA/SDTA851^e^ Mettler-Toledo (Greifensee, Switzerland). Samples were heated from 25 to 500 °C at a heating rate of 10 °C/min under a nitrogen flow of 60 mL/min.

Scanning Electron Microscopy (SEM)*.* Morphology of hydrogels was observed by LEO1450VP (LEO Electron Microscopy Ltd, Cambridge, England) scanning electron microscope operating at 20 kV. Prior to SEM, samples were freeze-dried in order to avoid the collapse of the porous structure in the SEM vacuum chamber, and sputter-coated with gold.

Mechanical Tests. Vacuum-dried hydrogels were cut in small stripes with a size of about 15 × 5 × 1 mm^3^ and the storage modulus at 1Hz was measured in the so-called “tension” configuration (extensional vibrations) as a function of temperature using a Perkin Elmer DMA8000 (Waltham, MA, USA). At the same frequency, the shear modulus was measured on pristine (immediately after synthesis) samples in the temperature range between 22 and 44 °C. In this case, samples had a cylindrical shape (5 mm diameter, 10 mm height) and were measured in the “shear” configuration (shear stress is applied at one end-face of the cylinder). Moreover, at room temperature the shear modulus was measured as a function of frequency.

Swelling Measurements. Samples taken from washed hydrogels were vacuum-dried for 24 h and then immersed in 0.5 M Na_2_SO_4_ aqueous solution, within a corked Falcon^®^ tube. The swelling ratios (SR %) of the hydrogels were calculated according to the expression:

(1) $$\text{SR}\;(\text{\%})=\frac{W_{s}-\;W_{d}}{W_{d}}\;\times \;\text{100}$$

where *W_d_* and *W_s_* are the weights of dry and swollen hydrogels, respectively. The swollen weight was taken on hydrogel wiped with moistened filter paper to remove excess liquid. 

Electrochemical Impedance Spectroscopy (EIS)*.* The ionic conductivity values of the hydrogels swollen for 24 h in electrolytic solutions were calculated from impedance spectra obtained by a Solartron Model 1255 frequency response analyzer (FRA) (Farnborough, Hampshire, UK). EIS spectra were recorded in the 1 kHz–1 Hz frequency range, with a signal amplitude of 5 mV under open-circuit conditions. Hydrogel samples and polytetrafluoroethylene (PTFE) spacers pierced at the core, to host the hydrogel, were sandwiched between two blocking electrodes (stainless steel disks) of a polyethylene cell, to assure a constant cell geometry. The spacer thickness was just lower than that of the hydrogel, in order to assure a good electrical contact, avoiding excessive deformation of the sample. The ionic conductivity of the swollen hydrogels has been obtained from the electrolyte resistance, achieved from a nonlinear fitting of the impedance spectra. Error on each conductivity value arises from the uncertainties on both the fitting procedure and geometrical factors of the samples.

Cyclic Voltammetry (CV). A T-cell was assembled with two platinum electrodes in contact with two disks of hydrogels swollen for 24 h in a 0.25 M K_4_[Fe(CN)_6_]3H_2_O/K_3_[Fe(CN)_6_] aqueous solution. A platinum wire embedded between the two hydrogel disks was used as pseudo-reference electrode. Cyclic voltammetries were recorded in the 0.6 V/–0.6 V potential *vs.* Pt range, at potential scan rates from 1 to 150 mV s^−1^, using a VSP BioLogic potentiostat. The diffusion coefficient D (cm^2^ s^−1^) of the redox couple Fe(CN)_6_^4−^/Fe(CN)_6_^3−^ has been calculated from the slope of the straight line obtained by plotting the peak current density against the square root of the potential scan rate, according to the Randles-Sevcik equation.

## 4. Conclusions

It has been demonstrated that the synthesis of cellulose hydrogels, with tunable characteristics of the final materials, can be successfully achieved. In doing this, the type of cellulose plays a key role: the synthetic strategy being equal, it is possible to control the relative extension of the physical and chemical crosslinking by using different starting celluloses and a convenient concentration of the crosslinking agent. This allows balancing of two competitive properties of the hydrogel, such as its liquid uptake and its mechanical features. It has been found that the synthesized cellulose hydrogels are able to absorb and retain, without leaks, a significant amount of electrolytic solution. The ionic conductivity measured on the hydrogels swelled in 0.5 M Na_2_SO_4_ aqueous solutions approaches that of the starting swelling electrolyte. Moreover, very high diffusivity and reversible redox behavior, evaluated by cyclic voltammetry tests on Fe(CN)_6_^4−^/Fe(CN)_6_^3−^-absorbed hydrogels, have been detected. Preliminary tests on the chemical stability of the hydrogels in acidic (pH 1) and alkaline (pH 12.7) aqueous solutions are in progress, with very promising results.

The data here shown clearly highlight that cellulose-based hydrogels, obtained by low-cost synthetic routes, have very appealing properties to be used as gel electrolyte membranes. Based on these preliminary results, a possible application in solid-state electrochemical devices, such as batteries, fuel cells, or electrolyzers, could be envisaged.
